# Allelic Imbalances in Radiation—Associated Acute Myeloid Leukemia

**DOI:** 10.3390/genes2020384

**Published:** 2011-05-31

**Authors:** Sergiy V. Klymenko, Jan Smida, Michael J. Atkinson, Volodymir G. Bebeshko, Michaela Nathrath, Michael Rosemann

**Affiliations:** 1 Institute of Clinical Radiology, Research Centre for Radiation Medicine, 53 Melnikova, 04050 Kyiv, Ukraine; E-Mails: klymenko_sergiy@yahoo.co.uk (S.V.K.); tayainna@mail.ru (V.G.B.); 2 Institute of Pathology, Helmholtz Zentrum Muenchen–German Research Center for Environmental Health, Ingolstaedter Landstrasse 1, 85764 Neuherberg, Germany;E-Mail: smida@helmholtz-muenchen.de (J.S.); 3 Institute of Radiobiology, Helmholtz Zentrum Muenchen–German Research Center for Environmental Health, Ingolstaedter Landstrasse 1, 85764 Neuherberg, Germany;E-Mail: atkinson@helmholtz-muenchen.de (M.J.A.); 4 Clinical Cooperation Group Osteosarcoma, Helmholtz Zentrum Muenchen, German Research Center for Environmental Health and Department of Pediatrics, Technische Universitaet Muenchen,Ingolstaedter Landstrasse 1, 85764 Neuherberg, Germany; E-Mail: Michaela.Nathrath@lrz.tum.de

**Keywords:** acute myeloid leukemia, ionizing radiation, Chernobyl accident, single nucleotide polymorphism, microarray

## Abstract

Acute myeloid leukemia (AML) can develop as a secondary malignancy following radiotherapy, but also following low-dose environmental or occupational radiation exposure. Therapy-related AML frequently carries deletions of chromosome 5q and/or 7, but for low-dose exposure associated AML this has not been described. For the present study we performed genome-wide screens for loss-of-heterozygosity (LOH) in a set of 19 AML cases that developed after radiation-exposure following the Chernobyl accident. Using Affymetrix SNP arrays we found large regions of LOH in 16 of the cases. Eight cases (42%) demonstrated LOH at 5q and/or 7, which is a known marker of complex karyotypic changes and poor prognosis. We could show here for the first time that exposure to low-dose ionizing radiation induces AML with molecular alterations similar to those seen in therapy-related cases.

## 1. Introduction

Ionizing radiation is established as a cause of leukemia in humans. Epidemiological life-span studies on the Japanese A-bomb survivors revealed that leukemia, primarily acute myeloid leukemia (AML), exhibits the highest excess relative risk of all neoplasms [1]. Epidemiological studies also identified a significantly increased leukemia incidence in clean-up workers at the Chernobyl Nuclear Power Plant [2,3]. The AML cases among Chernobyl clean-up workers and inhabitants of Ukrainian territories with high contamination from radioactive fallout were characterized by an unfavorable clinical course with resistance to conventional therapy. These patients had significantly shorter overall survival (OS), and lower complete remission rate as compared to sporadic cases [4]. The clinical observations suggest that the biology of the radiation-associated AML following the Chernobyl accident might be different from that of the spontaneous forms of AML. Cases of radio- and/or chemotherapy-related secondary AML are frequently found to carry chromosomal deletions at 5q and 7p/7q [5]. These recurrent alterations are related to complex karyotypic changes and bad prognosis. To understand the mechanisms of radiation leukemogenesis it would be important to determine if these 5q and 7p/7q deletions are only prevalent after high-dose radiotherapy, or if they also occur in AML associated with a preceding low-dose accidental or environmental radiation exposure.

The identification of molecular tumor markers that are associated with exposure to ionizing radiation could provide new insight into the pathogenesis of mutagen-induced AML. In a previous study we found that AML patients with a history of radiation exposure due to the Chernobyl accident exhibited AML-specific translocations affecting the *AML1* and *MLL* loci less frequently as compared to spontaneous or topoisomerase II inhibitor-related AML cases [6,7]. We have now expanded our investigations to include the analysis of the gains and losses of chromosomal material and the analysis of allelic changes or loss-of-heterozygosity (LOH), both to identify potential fingerprints of the radiation-etiology of this malignancy. We applied a SNP array based LOH screen that was recently shown to be a useful method to detect genomic alterations in AML [8].

## 2. Results and Discussion

Of the 19 radiation-associated AML cases analyzed in this study, 16 had at least one large region exhibiting allelic homozygosity ranging from 4.7 Mb up to 156.6 Mb somewhere in their genome. These regions were classified as areas of LOH (likelihood score ranged from 5 to 70) and are not known to be present in normal control genomes. Of these 16 LOH positive cases, 10 exhibited LOH in multiple regions of the genome (Table 1).

**Table 1.** Sample and chromosomal loss-of-heterozygosity (LOH) information of radiation-associated acute myeloid leukemia (AML) patients.

**Case**	**Blast cells in Bone Marrow Sample, %**	**LOH regions**			**Gains without LOH**

		**Loss**	**Copyneutral Aberration**	**Gain**	
1	90	5q21–5q34 7 12p12.3–12p13.31 19p12–19q13.11			19q13.41–19q13.43

2	20	5q14.1–5q34 7p21–7q36.1 12q14.3–12q23.3 16q12.1–16q22.1 16q23.1–16q23.1			

3	8	3p12.1–3pter 3q11.2–3q13.31 5q14.3–5qter 7p12.3–7p22 7q31.32–7q36.3 13q13.3–13q14.3		7q23.3–7q31.31 13q14.3–13q21.3	

4	66	5q14.2–5q15 5q23.2–5q31.3 5q33.2–5q33.3 13q14.1–13q14.11	2q12.1–2q12.3 7p14.3–7p14.1 15q11.2–15qter 16q13–16q22.1 16q22.2–16q23.2	13q2.33–13q31.3	

5	82		1p14–1p12		

6	100		4q26–4q28.1		

7	61		12qcen–12q12.1		8p11.21–8q24.3

8	20		4q26–4q28.1 7p11–7qter 11q12.1–11q12.3 14q13.2–14q21.3		

9	44				

10	66		6p22.2–6p21.31 9p13.1–9q22.32 14q24.3–14q31.2		

11	93		6p25.1–6p24.1		

12	95	5q21–5q33.1 17q11.2–17q25.3	11p11.12–11p11.2		8p11.21–8q24.3

13	97		11p11.12–11p11.2		

14	58	7	2q12.3–2q14.3		

15	32				

16	60	5q14.1–5q22.1 5q23.2–5qter	1p36.11–1p34.3 6q15–6q16.1 8p22–8pter		8p11.21–8q24.3

17	77				

18	70		3p26–3p24.3 3q26.31–3q26.33		

19	100		21q11.2–21qter		13q22.1–13q34

**Figure 1:**
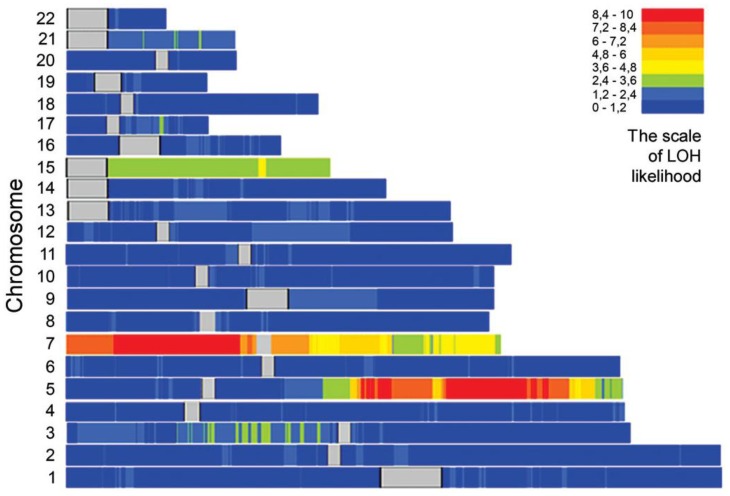
The pattern of genome-wide LOH score derived by SNP arrays in set of 19 radiation-associated AML samples. The average of LOH-likelihood values ranges from blue (lowest probability) to red (highest probability). Centromeres are indicated with grey. The high LOH-score at chromosome 15 was due to homozygosity along the entire chromosome in a single case.

The overall distribution of positioning of the LOH in the genome (Figure 1) clearly exhibits a non-random distribution, with chromosome 5q and chromosome 7 contributing most frequently to the overall LOH frequency. Abnormalities at 5q, 7p or 7q were found in 8 patients (42%), which is significantly more frequent than in AML cases without a preceding radio- or radio-chemotherapy analyzed with an identical method [8].

In four of the eight cases with losses affecting chromosome 5q or 7, the LOH was found concordantly at both chromosomes. Of all these 12 individual chromosomes with 5q and/or 7p/7q LOH, 10 were associated with reductions in DNA copy numbers. This suggests that SNP LOH is a useful surrogate for the 5q and 7p/7q losses originally found by comparative genomic hybridization (CGH).

Additional recurrent LOH loci were found on 4q and 11p (2 cases each with copy-neutral changes), 13q (1 case with copy-number loss, 2 cases with gains of DNA), 16q and 3p (1 case each with copy-neutral change, 1 case each with copy-number loss) (Table 1).

AML in patients with LOH at chromosome 5q and/or 7 was more often preceded by myelodysplastic syndrome (MDS) than in cases without any of such abnormalities (*r_s_* = 0.57, *p* = 0.011). There were no correlations between the presence of LOH at chromosome 5q and/or 7 and age of patient, age above 60, higher white blood cells count, white blood cells above 30 × 10^9^/L at presentation, or *FLT3* gene mutation status. Interestingly, patients with LOH at either of the chromosome 5q or 7 had higher white blood cells count (*r_s_* = 0.87, *p* = 0.005) as compared to patients with combined LOH at chromosome 5q and 7.

The functional relevance of LOH in the pathogenesis of acute leukemia remains a controversial issue. Whereas genome-wide analysis on childhood AML reported LOH somewhere in the genome in 32% of cases [9], a much higher rate was found in adult leukemia [10]. In the present study of adults we found clear indications for recurrent LOH at 5q, and 7 in radiation-associated AML. In most cases the detection of LOH at these loci was associated with loss of genomic material, implying that the former resulted from interstitial deletions or losses of entire chromosome arms. Some of copy-neutral allelic losses could be due to somatic uniparental disomia as reported in AML [11].

A concordant loss at 5q and 7 was recently reported to characterize AML with complex karyotypic alterations and unfavorable prognosis [8,12,13]. A similar bad prognosis has been found for cases with deletions of the long arm or the entire chromosome 7 only [14]. It should be noted that, based on studies on AML without a preceding radiation or other mutagen exposure [13,15], we would expect only about three cases with losses and deletions at chromosomes 5q and/or 7. Therefore, the frequency of eight AML patients in our study group carry this alteration is significantly higher than expected (*p* = 0.003, assuming a binomial distribution), but would be in agreement with the above estimation that only one third of our cases are really caused by the radiation exposure. The observation that the relative excess of cases showing 5q and/or 7 aberrations is much higher than the relative excess of all AML cases due to radiation exposure suggests, that ionizing radiation not simply causes an increase in AML by number, but that it also changes the spectrum of AML types towards those with a less favorable prognosis.

We reason that the unusual high prevalence of 5q and 7 alterations in our patient cohort has to be attributed to their unique etiology, with external total-body irradiation resulting from the Chernobyl accident. The average external dose to the entire patient cohort multiplied by the excess relative AML risk per unit dose as derived from epidemiological studies [1–3] would suggest that roughly one third of our cases have a radiation etiology, whereas the rest developed spontaneously. It is also worth noting that in contrast to data from spontaneous AML cases, we observed 5q and/or 7 allelic losses equally frequent in younger and in older patients (Table 1). This would suggest that the genotoxic effects of ionizing radiation accelerate the pathogenic process of AML such that complex karyotypic alterations, usually arising only later in life contribute to the leukaemogenesis in younger patients as well.

The patients in our study, that have been accidentally exposed to rather low doses of ionizing radiation, exhibit a similar pattern of LOH/losses at chromosomes 5 and/or 7 as compared to persons developed AML following chemotherapy with alkylating agents alone or in combination with high-dose radiotherapy, where losses of chromosomal material at chromosomes 5 and/or 7 are of major importance [16]. The patients included in our study did not experience chemo/radiotherapy or any other known mutagenic exposure except low-dose ionizing radiation due to the Chernobyl accident. This suggests that high and low doses of ionizing radiation induce AML with the same pattern of genetic alterations. Because of its clinical relevance, this finding might create the necessary prerequisites to give a more precise definition to mutagen-induced cases of AML in classification of neoplasms.

In summary, the use of a high-resolution SNP mapping technique revealed a high frequency of LOH at 5q and 7 in patients with radiation-associated AML following the Chernobyl accident. LOH at 5q and/or 7 was present in 42% of all radiation-associated AML cases and was associated with poor prognosis. The lack of matched normal DNA samples most likely hindered the possibility to identify small regions of LOH. Therefore, the real prevalence of these abnormalities in Chernobyl AML patients might be even higher than that detected in the present study.

## 3. Experimental Section

### 3.1. Clinical Samples

The study group consists of 19 adult AML patients, initially diagnosed between 1995 and 2004 and treated with standard chemotherapy according to European Society of Medical Oncology Minimum Clinical Recommendations effective at the time of diagnosis. They were recruited for the study in accordance with the principles of the Helsinki Declaration after the approval of the study by the local Ethics Committee (Research Centre for Radiation Medicine, Kyiv, Ukraine). Details of patients' age, sex, preceding MDS, French-American-British (FAB) type, and available cytogenetic and molecular genetic information are given in Table 2. The sole criterion for inclusion of the patients into this study was the suitability of biological material for the chosen assay. Thirteen of the patients were involved in clean-up operations at the Chernobyl Nuclear Power Plant in 1986–1987 with one of which survived 2nd degree acute radiation syndrome (case 10), one patient was evacuated from the Chernobyl exclusion zone and five were resident in Ukrainian territories with high levels of contamination from radionuclide fallout. The average effective dose from external irradiation was estimated to be 130 mSv for clean-up workers [17] and 15–17mSv for cases from contaminated areas [18,19].

### 3.2. SNP Chip Assay

Genomic DNA was extracted using the QIAamp Mini DNA extraction kit (Qiagen, Hilden, Germany) from bone marrow samples obtained at diagnosis and preserved frozen at −70 °C.

DNA samples were processed according to the GeneChip Mapping 10K (V2.0) *Xba* Assay protocol (Affymetrix Inc., Santa Clara, CA, USA). Briefly, 250 ng of DNA was digested with *Xba*I and ligated to the *Xba*I adaptor prior to polymerase chain reaction (PCR) amplification using AmpliTaq Gold (Applied Biosystems, Foster City, CA, USA) and primers that recognize the adapter sequence. The amplified DNA was fragmented, end-labelled with a fluorescent tag and hybridized to the array. Hybridized arrays were processed with an Affymetrix Fluidics Station 450 and fluorescence signals were detected using the Affymetrix GeneChip Scanner 3000. On average, 96.7% (range 92.6% to 98.2%) of all SNPs could reliably be genotyped, resulting in more than 10,000 informative SNPs per case with an estimated mean distance between consecutive markers of about 279 kbp.

**Table 2.** Anamnestic, clinical, cytogenetic and molecular genetic information of radiation-associated AML patients.

**Case**	**Group**	**Sex/Age,Years**^a^	**Occupation**	**Preceded by MDS**	**FAB Type**	**Latency Time,Years**^b^	**Karyotype**	***AML1/ETO* by FISH or RT-PCR**	***MLL*translocation**			***FLT3*ITD**	***FLT3* D835**
									**FISH Results**	**RT-PC Results**			
										***MLL/AF9***	***MLL/AF4***		
1	CW	M/60	engineer	Yes	M0	16	ND	negative	negative	ND	ND	WT	WT
2	CW	M/73	economist	Yes	M2	17	ND	ND	ND	negative	negative	WT	WT
3	CW	M/42	driver	Yes	M6	17	ND	negative	ND	ND	negative	WT	WT
4	CW	M/42	welder	Yes	M6	9	ND	ND	ND	ND	ND	WT	WT
5	CW	M/67	sailor	No	M0	18	ND	ND	ND	ND	ND	WT	WT
6	CW	M/59	agronomist	No	M1	16	ND	negative	negative	negative	negative	WT	WT
7	CW	M/55	farmer	No	M2	18	47,XY, +8[12]	negative	negative	ND	ND	WT	WT
8	CW	M/66	policeman	No	M2	16	ND	negative	negative	ND	negative	Mut	WT
9	CW	M/76	ND^c^	No	M2	15	ND	negative	negative	ND	ND	WT	WT
10	CW	M/59	security guard	No	M4	11	ND	negative	negative	ND	ND	WT	WT
11	CW	M/62	serviceman	No	M4	16	46,XY [15]	negative	negative	negative	ND	WT	WT
12	CW	M/35	driver	No	M5a	11	ND	negative	negative	ND	ND	Mut	WT
13	CW	M/43	loader	No	M5a	17	46,XY [15]	ND	negative	ND	ND	Mut	WT
14	V	M/29	mechanic	Yes	M4	16	ND	negative	negative	ND	ND	WT	WT
15	V	M/57	mechanic	Yes	M5a	16	ND	negative	negative	ND	ND	WT	WT
16	V	M/71	farmer	No	M0	14	ND	negative	negative	ND	ND	WT	WT
17	V	F/26	housewife	No	M1	17	ND	negative	negative	ND	ND	WT	WT
18	V	M/33	tractor driver	No	M4	12	ND	negative	negative	ND	ND	WT	WT
19	V	M/42	crane-operator	No	M5b	15	ND	negative	negative	ND	ND	WT	WT

MDS: myelodysplastic syndrome; FAB: French-American-British; FISH: fluorescence *in situ* hybridization; RT-PCR: reverse transcription polymerase chain reaction; *FLT3* ITD: internal tandem duplications of *FLT3* gene status by PCR; *FLT3* D835: *FLT3* D835 mutation status by PCR; CW: clean-up worker of the Chernobyl accident; V: victim, indicates patient evacuated from the Chernobyl exclusion zone or domiciled in highly contaminated with radioactive fallout rural areas of the Ukraine; M: male; F: female ND: no data; WT: wild type; Mut: mutated;

a : at time of diagnosis;

b : time since first exposure due to the Chernobyl accident to overt AML;

c : retired at the time of diagnosis.

### 3.3. Data Analysis

The primary hybridization data were processed using Affymetrix GTYPE software, yielding the genotype and hybridization intensity for each individual SNP marker. In a second step Affymetrix^®^ GeneChip^®^ Chromosome Copy Number Analysis Tool (CNAT) was applied to detect genomic regions with unusual long stretches of contiguous homozygote SNPs. Such uninterrupted areas of homozygosity are assigned a likelihood score that marks them as potential a somatic LOH [20]. To distinguish tumor-specific somatic LOH from potential germline homozygosity, a cut-off LOH score of 5 was used. This was equivalent to a contiguous homozygout interval of at least 4.7 Mbp in length. Since a homozygous interval of this length was not observed in the genome of more than 100 healthy donors (reference data provided by Affymetrix), we could assume that it truly represented a tumor-specific somatic LOH.

For graphical presentation of copy number alterations and LOH the data were finally imported into ArrayCGHbase software tool [21].

### 3.4. Statistical Analysis

Correlation analysis was performed using Spearman's rank order correlation coefficient (*r_s_*). Differences were considered significant at *p* < 0.05. The statistical analyses were done with STATISTICA 4.5 (StatSoft, Tulsa, OK).

## 4. Conclusions

External exposure to low doses of ionizing radiation induces AML with a similar pattern of chromosome 5q and 7p/7q alterations as in high-dose therapy-related AML.

We hypothesize that LOH at chromosome 5q and/or 7 as consequences of copyneutral aberrations or due to allelic imbalances constitutes an important genetic mechanism involved in tumorigenesis following accidental radiation exposure at low doses. The relative excess of AML cases with 5q and/or 7 alterations in patients with a preceding radiation-exposure suggests that exposure to ionizing radiation not only increases the incidence of leukaemia, but that it changes the spectrum of AML towards types with less favorable prognosis.
